# *Aggregatibacter actinomycetemcomitans* induces biofilm formation of *Streptococcus sanguinis* on titanium implants

**DOI:** 10.1186/s40729-025-00616-8

**Published:** 2025-04-03

**Authors:** Andrea Schubert, Jana Marisa Friebel, Oskar Bunz, Christoph Sasse, Ralf Bürgers, Torsten Wassmann

**Affiliations:** Department of Prosthodontics, University Medical Center Goettingen, 37099 Goettingen, Germany

**Keywords:** *Aggregatibacter actinomycetemcomitans*, *Streptococcus sanguinis*, Biofilm formation, Titanium implant, Surface roughness

## Abstract

**Purpose:**

This study aims to investigate the distinct behaviors of single-species and dual-species biofilms formed by *Streptococcus sanguinis* and *Aggregatibacter actinomycetemcomitans* on different titanium and implant surfaces. Four types of surfaces were examined: two clinically used implant surfaces, a super-polished surface and a sand-blasted surface of grade 4 titanium.

**Methods:**

Specimens were incubated with single- and dual-species biofilms for 24 h. Biofilm formation was determined based on the amount of total DNA extracted from the bacteria. In order to specifically determine the biofilm formation of *Streptococcus sanguinis*, qPCR experiments were carried out. Staining followed by fluorescence microscopy was employed to verify the efficiency of the washing steps.

**Results:**

Biofilm formation by single- and dual-species cultures was observed on all tested implant surfaces. However, a clear influence of surface characteristics on biofilm formation could not be conclusively demonstrated. Mixed cultures of *S. sanguinis* and *A. actinomycetemcomitans* (*AAC*) exhibited increased biofilm formation through the enhanced DNA amount of *S. sanguinis*. In contrast, this effect was not observed in dual-species cultures of *Staphylococcus epidermidis* and *S. sanguinis*.

**Conclusion:**

*AAC* promotes biofilm formation of *S. sanguinis*, highlighting the significant role of *AAC* in enhancing biofilm development. Conversely, a definitive conclusion regarding the correlation between titanium implant surface roughness and biofilm formation was not possible. However, our results suggest a tendency that dual-species biofilm formation may be influenced by surface structure.

## Background

Peri-implantitis describes the inflammation of the tissues adjacent to osseointegrated implants that results in the loss of peri-implant bone tissue [1, 2]. Peri-implantitis shows high prevalence and is considered a major cause of implant loss [2, 3]. Microbiologically, it represents a biofilm-associated disease with a variable microbial spectrum [4, 5]. For preventive considerations the minimization of microbial adhesion on implant materials is desirable. In addition to the composition of the implant material, microbial adhesion depends on surface charge, surface free energy (SFE) and surface roughness [6–8].

Ti-6Al-4 V is a titanium alloy commonly used in dental implantology as an implant material per se and as a material for prosthetic abutments with evidence-based long-term reliability [9]. Sand-blasted and acid-etched surface modifications are used to optimize osseointegration, resulting in average mean roughness values (R_a_) of 1 to 2 μm [10]. The adhesion of biofilms to titanium surfaces with different roughness values has been investigated in vitro and in vivo: above an R_a_ threshold of 0.2 μm adhesion correlates with increasing roughness [11, 12]. In the pathogenesis of peri-implantitis, gram-positive early colonizers of oral surfaces such as *S. sanguinis* are considered non-pathogenic, whereas gram-negative species such as *AAC* and *Eikenella corrodens* seem to be associated with peri-implant inflammation [13–15]. The adhesion behavior of different microorganisms in monospecies biofilms on dental materials differs: the adhesion of *Candida albicans* showed a stronger positive correlation with increasing roughness values than *S. sanguinis* [16]. Little is known about how the adhesion behavior of different microorganisms in a multi-species biofilm changes depending on surface roughness. A changed biofilm composition may influence the pathogenicity with regard to the development of peri-implantitis. Although there is evidence that implant surfaces with low roughness do not prevent peri-implantitis [17], investigations on the influence of implant surfaces on peri-implant diseases are scarce [18].

The present in vitro study investigated the proportionate composition of dual-species biofilms from a non-pathogenic (*S. sanguinis*) and *AAC*, a microorganism that is often associated with peri-implantitis [19, 20], on titanium surfaces with different surface treatments and correspondingly different surface roughness. The results of the present study make a basic scientific contribution to the knowledge about biofilm formation on dental implant surfaces.

## Materials and methods

### Specimen preparation

Cylindrical specimens (10 mm x 2 mm) of four titanium materials with different surface specifications were included in the present study: super-polished specimens of grade 4 titanium (‘super-polished’), sand-blasted specimens of grade 4 titanium (‘sand-blasted’) and specimens of two commercially available and clinically used titanium implant surfaces (‘implant surface I’: Myplant Two titanium implants, Hager & Meisinger GmbH, Neuss, Germany; ‘implant surface II’: Semados titanium implants, BEGO GmbH & Co. KG, Bremen, Germany).

Super-polished specimens were manufactured as follows: rods of grade 4 titanium (Hempel metals and more, Duebendorf-Zurich, Switzerland) were sliced into disks using a separating machine (Micracut 201, Metkon, Bursa, Turkey) and polished with an automated grinding machine (Digiprep 251, Metkon) and silicon carbide grinding paper with decreasing grain sizes (400, 800, 1200, 4000), then polished using polishing papers with grit sizes of 3 μm and 1 μm. Sand-blasted specimens were obtained likewise, but sand-blasted instead of polished using an aluminum oxide grit size of 110 μm (Alublast 110 μm, Henry Schein Inc., Melville, USA) for 6 s at a pressure of 1 bar and at a distance of 100 mm using a sandblasting device (Basic Master, Renfert GmbH, Hilzingen, Germany). Specimens of implant surface 1 and implant surface II were provided by the respective implant manufacturers.

### Surface characterization

Mean surface roughness R_a_ was calculated for five specimens of each material via confocal microscopy (Zeiss Smartproof 5, Carl Zeiss Microscopy, Jena, Germany) and automated software analysis (Confomap, Carl Zeiss Microscopy). The SFE was determined as described before [16]. In brief, contact angle measurements were performed for each tested material: one µl of distilled water and methylene iodide, respectively, was applied to the specimen’s surface. Within 30 s after application, a computer-aided measurement device (Drop Shape Analyzer DSA25, Krüss, Hamburg, Germany) performed ten contact angle measurements for each liquid. The SFE was calculated using the formula introduced by Owens and Wendt [21].

### Scanning electron microscopy

The imaging of the test specimen surfaces was performed using a scanning electron microscope (JEOL JSM-IT500 InTouchScope, JEOL Ltd., Akishima, Japan) at a voltage of 20 kV. For each test material, one representative area with and without bacterial colonization was captured at a magnification of 2700x.

### Microbial adhesion

*S. sanguinis* (lot nr. 20068, Deutsche Sammlung von Mikroorganismen und Zellkulturen DSMZ, Braunschweig, Germany), *AAC* (lot nr. 8324, DSMZ) and *Staphylococcus epidermidis* (lot nr. 8340, DSMZ) were cultured in liquid Brain Heart Infusion Broth medium (lot nr. CM1135, Oxoid Limited, Hampshire, UK) at 37 °C. Microorganisms were harvested by centrifugation, washed twice with phosphate-buffered saline (PBS, Merck, Darmstadt, Germany) and resuspended in PBS. For experimental procedures, suspensions in PBS with an adjusted optical density of 0.2 at 600 nm were prepared by densitometry (Bio Photometer, Eppendorf, Hamburg, Germany). Under sterile conditions, specimens of the test materials were transferred to 24-well plates and attached to well bottoms using silicone (Z-Dupe, Henry Schein Dental, Langen, Germany). For the mono-species biofilms, 1 ml of *S. sanguinis*, *S. epidermidis* or *AAC* suspension was added to each well and incubated for 24 h at 37 °C and 55 rpm.

In order to obtain a similar bacterial density as in the single culture, the amount was halved in the two-species biofilm and later adjusted mathematically in the analysis. For the dual-species biofilms, 500 µl of each bacterial suspension was added to the well, mixed using a sterile pipette (Sarstedt AG & Co. KG, Nürnbrecht, Germany) and incubated under the previously described conditions.

### Fluorescence staining

In order to visualize the bacterial biofilms on the test surfaces, fluorescence staining was performed using the DNA dye Hoechst 33,342 (Sigma-Aldrich, St. Louis, USA). Following the incubation, specimens were washed three times with 0.85% saline; 1 mL bisbenzimide H 33,342 trihydrochloride (Sigma Aldrich, Munich, Germany) was added to each specimen for 10 min. Staining solution was removed via three washing steps with 0.85% saline, and microbial cultures were fixated using 8% paraformaldehyde solution. After 10 min, specimens were dried and mounted on object slides for visualization via fluorescence microscopy (BZ-X710, Keyence, Osaka, Japan).

### Biofilm removal and DNA isolation

The biofilm removal and isolation of bacterial DNA was performed using the QIAamp DNA Mini Kit (Qiagen N.V., Venlo, Netherlands). After incubation, the bacterial suspension was removed, specimens were washed with 1 ml 0.85% saline and transferred to sealable reaction tubes (Eppendorf, Hamburg, Germany). The cell lysis and isolation of the DNA was conducted in accordance with the specifications provided by the manufacturer. The removal of biofilms was confirmed by repeated fluorescence staining following the described protocol.

### Primer designing and real-time PCR

Specific oligonucleotide primers were identified for use in real-time PCR with the selected bacterial strains based on literature research in the *N*ational *C*enter for *B*iotechnology *I*nformation (NCBI) database. The sensitivity and specificity of the identified primer sequences were assessed using the Basic Local Alignment Search Tool (BLAST, http://blast.ncbi.nlm.nih.gov/Blast.cgi) in an in-silico PCR. Primer sequences lacking specificity for a certain bacterial strain or cross-reactivity with two bacterial strains were excluded. After a data-based review of the sequences, a Genomics product manufacturer (Eurofins Genomics, Ebersberg, Germany) produced an individual primer for each bacterial species (Table 1).

qPCR experiments were performed in a Mic qPCR Cycler (Bio Molecular Systems, Upper Coomera, Australien) according to the standard protocol. Briefly, a denaturation step at 94 °C after an initialization step at 95 °C was used. The denaturation temperature was 57.5 °C, based on the primer sequence. A total of 30 cycles were used for the analysis. Primers specific for *S. sanguinis* were used for all qPCR experiments (Table 1). A SYBR Green mix (Kapa SYBR FAST, Roche Holding AG, Basel, Schweiz) was used according to the user’s manual. Data analysis was performed using the Mic qPCR Analysis Software (Bio Molecular Systems, Upper Coomera, Australien). Five biological replicates were performed. After the real-time PCR, an exemplary reaction product for each primer was sterilely stored at -18 °C and sent to an external analysis laboratory for DNA sequencing.

**Table 1:** Overview of oligonucleotide primers (target species and sequence of the species-specific forward (F) and reverse primer (R).)

Target species	primer	primer sequence
*S. sanguinis*	S2	F: CAA AAT TGT TGC AAA TCC AAA GG R: GCT ATC GCT CCC TGT CTT TGA

### Statistical analyses

**Fig. 1:**
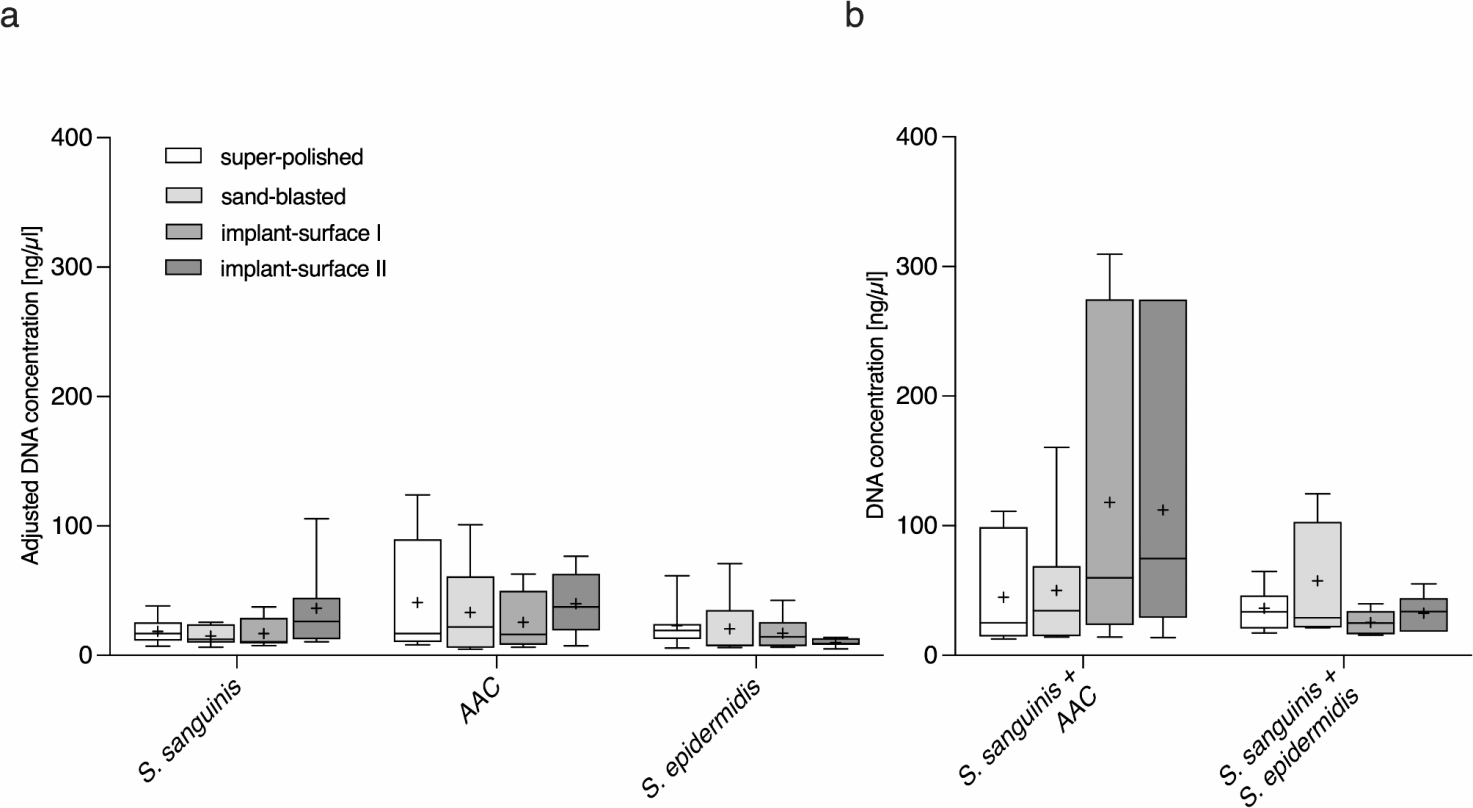
DNA concentration of the investigated biofilms on varying test materials. (**a**) Adjusted (halved) single-species biofilms and (**b**) measured DNA concentrations of two-species biofilms. The + marks mean values, 75% quartiles, median and 25% quartiles are shown

The adhesion experiment data, including DNA concentrations, are presented as boxplots with 75% quartile, median and 25% quartile. To ensure a comparable representation of DNA concentration, the values for the single-species biofilms were adjusted accordingly. During the experiment, the bacterial strains in the two-species biofilms were halved to achieve a bacterial density comparable to that of the single-species biofilms. To account for these differences in bacterial quantities, the results were also halved for Fig. 1a and labeled as “adjusted values.”

The qPCR evaluation comparing dual-species biofilms of *S. sanguinis* with *AAC* or with *S. epidermidis* were assessed for normal distribution using a Q-Q plot and then analyzed using a two-way ANOVA with Šídák’s multiple comparisons test, with an alpha level of 0.05.

## Results

### Surface characteristics

The R_a_ values of the examined specimens ranged from 0.062 μm (± 0.023, super-polished) to 1.405 μm (± 0.093, implant surface I). SFE values varied from 55.430 mN/m (± 2.861, super-polished) to 65.214 mN/m (± 4.577, implant surface I) (Table 2). No correlation was found between surface roughness and SFE (data not shown).

**Fig. 2:**
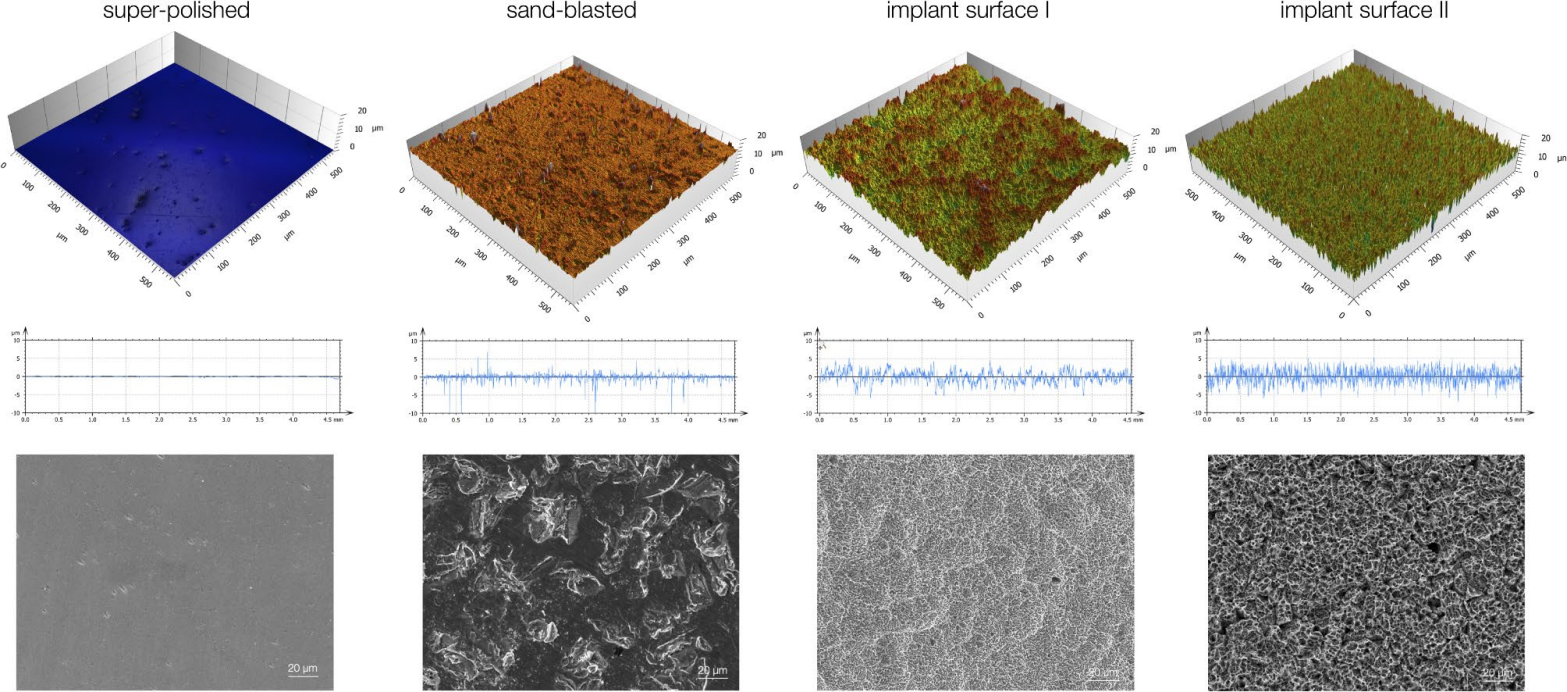
Surface characteristics of the tested materials. 3D representation of the surface topography (upper row), 2D representation of the roughness profile (middle row), scanning electron microscopy (bottom row). The super-polished surface appears homogenous, the sand-blasted surface and implant surfaces I and II are irregular

Figure 2 provides an overview of the 3D representation of the surface topography, the 2D representation of the roughness profiles and the corresponding scanning electron microscopy images. Super-polished specimens had a macroscopically smooth appearance corresponding to a low R_a_ value, while the higher peaks in the profiles of the other materials reflected higher R_a_ values. The surface of the sand-blasted group showed occasional very high peaks in both the three-dimensional representation and the two-dimensional profile curve.

The scanning electron microscopy images unveiled that the super-polished sample group had a homogeneous surface without grooves or ridges (Fig. 2). The sand-blasted sample group appeared rugged and rough due to the impacts of the blasting material, with the structuring of the sand-blasted surface appearing significantly coarser compared to implant surfaces I and II. In addition to coarse inhomogeneities, the surfaces of implant surfaces I and II exhibited a finely rugged surface structure, possibly due to the two-step surface treatment involving sandblasting and acid etching. Compared to implant surface II, implant surface I had a similar but finer surface structure.

**Table 2:** Mean values and standard deviations of R_a_ and SFE values of the tested materials

Test material	*R*_a_ [µm]	Surface free energy [mN/m]
super-polished	0.062 (± 0.023)	55.43 (± 2.86)
sand-blasted	0.505 (± 0.232)	63.93 (± 1.96)
implant surface I	1.325 (± 0.138)	65.21 (± 4.58)
implant surface II	1.405 (± 0.093)	59.01 (± 2.93)

### Biofilm formation

**Fig. 3:**
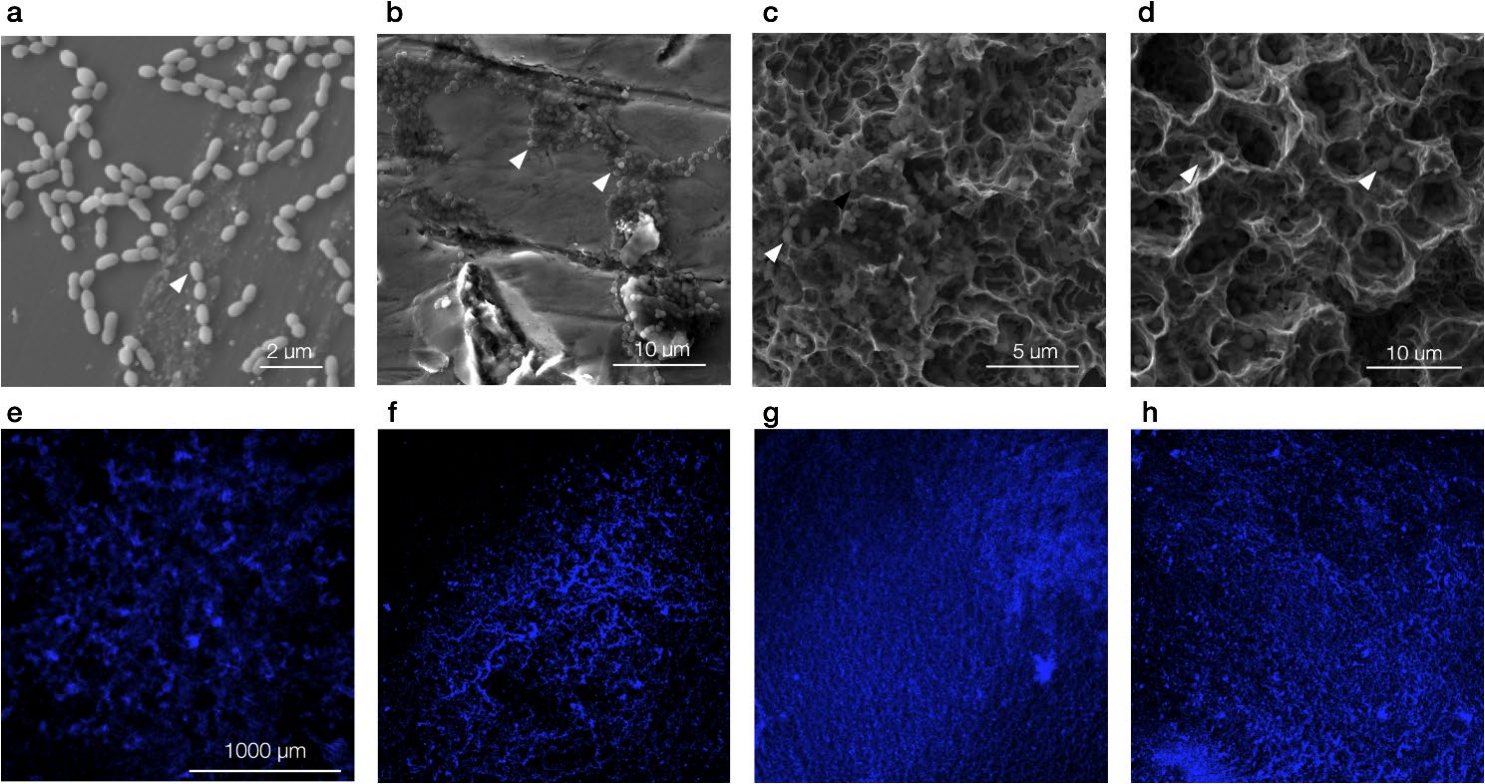
Biofilm formation on the tested materials. Scanning electron microscopy (**a**-**d**) and fluorescence staining (**e**-**h**) of investigated biofilms on different test materials S. sanguinis on the super-polished surface (**a**), S. epidermidis on the sand-blasted surface I (**b**), S. sanguinis with AAC on implant surface I (**c**), S. sanguinis with *S. epidermidis* on implant surface II (**d**), AAC on the super-polished surface (**e**), S. sanguinis on implant surface I (**f**), *S. epidermidis* on implant surface II (**g**), *S. sanguinis* with AAC on implant surface II (**h**)

In order to find out whether the surface roughness of titanium implants has an influence on biofilm formation with various bacteria, corresponding investigations were carried out. Therby two typical oral bacteria species (*AAC* and *S. sanguinis*) and one non typical oral bacterial species (*S. epidermidis*) were used. Fluorescence staining and scanning electron microscopy were performed to visualize the adhesion of mono-species and dual-species to the tested materials. Exemplary images are shown in Fig. 3. The cells of *S. sanguinis* were organized in typical chains, while the cells of *AAC* were arranged in pairs or clusters. The cells of *S. epidermidis* formed clusters on the surface.

The concentration of isolated DNA was used for determination of the biofilm formation. Different colonization behaviour was observed in the tested bacteria, with the strongest biofilm formation being measured for *AAC*. However, no clear differences were found between the used titanium surfaces with regard to colonization of the various individual bacterial cultures (Fig. 1a). In contrast, the combination of *S. sanguinis* and *AAC* showed a higher amount of DNA on impant-surface I and II compared to the single cultures, whereas the co-cultivation of *S. sanguinis* and *S. epidermidis* did not lead to this strong tendency (Fig. 1a and b). DNA quantity of the mixed culture of *S. sanguinis* and *S. epidermidis* appears to correspond somewhat to the sum of the quantities of the corresponding individual cultures. Nevertheless, no significant differences could be observed. Based on these results the surface roughness of titanium implants appears to have no effect on biofilm formation under the tested conditions. In contrast, the composition of the bacterial cultures seems to influence directly the biofilm formation (Fig. 1b).

### Quantification of *S. sanguinis* in two-species biofilms

**Fig. 4:**
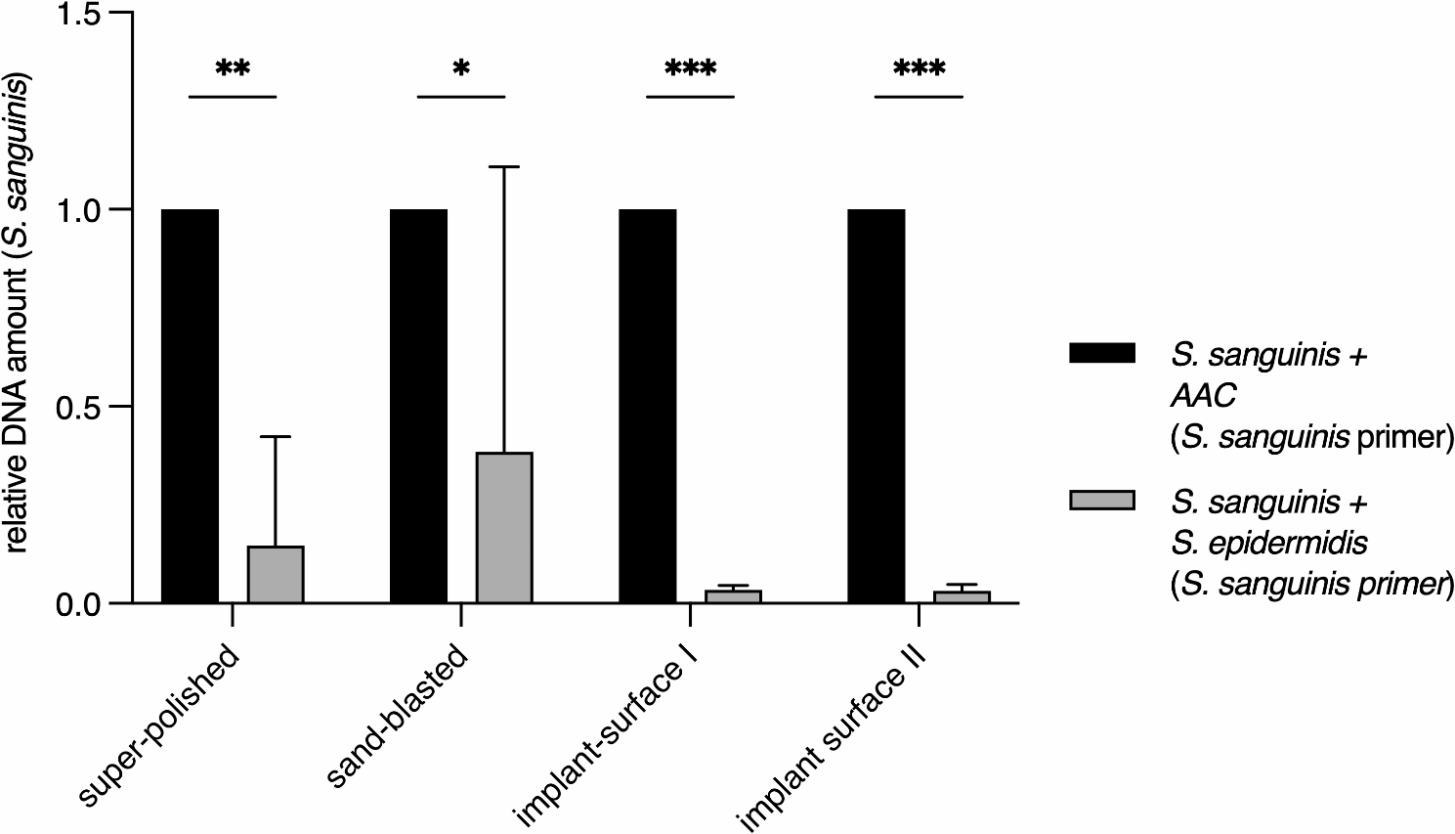
AAC increases the biofilm formation of *S. sanguinis* on the tested materials. Relative DNA amount of *S. sanguinis* for two-species biofilms on tested titanium surfaces, using *S. sanguinis-specific* primers. Samples with AAC and S. sanguinis were normalized to 1. Five biological replicates were used for analysis. Error bars indicated the standard deviation (SD). Significant differences between biofilm groups are indicated by asterisks (* = *p* ≤ 0.05; ** = *p* ≤ 0.001; **0 = *p* ≤ 0.0001)

We wanted to find out whether the biofilm formation of *S. sanguinis* can also be increased depending on certain microorganisms. Previous work has shown that co-cultivation of *S. mutans* and *AAC* increases biofilm formation [22], whereas a similar effect has not been described for *S. epidermidis*. By using *AAC* and *S. epidermidis*, it should be found out whether an increased biofilm formation of *S. sanguinis* can be induced in dependency of *AAC*. *S. epidermidis* was used as control. Therefore, the relative amount of *S. sanguinis* within dual-species biofilms, consisting of *AAC* or *S. epidermidis*, was investigated by qPCR (Fig. 4). Therefore, *S. sanguinis* specific primers were used (see method part). Five biological replicates were performed and data were statistical anaylsed using the two way ANOVA.

The two-species biofilm consisting of *S. sanguinis* and *AAC* shows that *S. sanguinis* was significantly more present in comparison to the mixed culture of *S. sanguinis* and *S. epidermidis*. This effect could be oberserved for all tested material surfaces (Fig. 4).

These data implies that *AAC* but not *S. epidermidis* supports biofilm formation of *S. sanguinis* on surfaces of titanium dental implants independent of the R_a_ value of the surface.

## Discussion

A critical point in the pathogenesis and etiology of peri-implant disease is the initial adhesion of bacteria [4, 5]. While the microbiome on an implant surface with healthy peri-implant tissue resembles a biofilm on the natural tooth substance, peri-implant lesions are dominated by gram-negative, parodontopathogenic pathogens such as *AAC*, *P. gingivalis* or *T. denticola* [13–15, 22]. Thus, the species-specific composition of peri-implant biofilms is of crucial importance in the etiology of peri-implant diseases.

Peri-implantitis normally occurs under anaerobic conditions. In this study, the biofilm experiments were carried out under aerobic conditions. This was done to obtain a simple, robust set up and to minimize deviations that are more likely to occur under anaerobic conditions. Of course, this can have an influence on biofilm formation. However, our results show that *AAC* increases biofilm formation in *S. sanguinis*, which is consistent with the data from Szafrański et al. [23]., where the experiments were performed in presence of CO_2_.

The mechanisms leading to the specific dominance of certain potentially pathogenic species are not yet understood. In this study we investigated titanium implants, which are still considered the gold standard in dental implantology [10, 24, 25], with regard on biofilm formation based on roughness and bacterial species.

The R_a_ value has already been discussed in various studies as a significant influence factor on bacterial adhesion [12, 26, 27], whereby surfaces with a higher R_a_ value have an increased potential for bacterial adhesion [12, 28]. In this work, a tendency was observed that biofilm formation of single-species cultures is independent of the surface roughness. This would partially explain why biofilms usually consist of several different microorganisms to increase the biofilm formation on different surfaces. This hypothesis is supported by our observations where we were able to recognize a dependency on the surface quality based on the measured DNA quantity for the mixed cultures. The biofilm formation on the implant-surface I and II, indicated by a higher amount of total bacterial DNA, seems to be increased in the mixed cultures of *AAC* and *S. sanguinis* than in the single cultures. This effect was not observed in the co-cultures of *S. sanguinis* and *S. epidermidis*. Therefore, biofilm formation seems to be more dependent on the bacterial composition than on the implant surface (Fig. 1b). Thus, cross-talk between different microorganisms from the same environment enables efficient colonization. However, this effect could not be confirmed by qPCR analysis of *S. sanguinis*. The data showed no significant increase in the amount of *S. sanguinis* in the mixed cultures with the different implants. Based on the current results, it is therefore not possible to make a definitive statement regarding biofilm formation on titanium implants in dependency on the R_a_ value. In future, further experiments are required in which bacteria from the oral microbiome are incubated in various combinations with titanium implants in order to gain a better understanding of oral biofilm formation in correlation with the R_a_ value.

The combination of *S. sanguinis* and *AAC* increases the biofilm formation in comparison to the corresponding single-species cultures. A similar effect was already described for *AAC* and *S. mutans* [23]. They could show that *AAC* activates quorum sensing (QS) in *S. mutans* by inducing the expression of the alternative sigma-factor encoding gene *sigX* which results in increased biofilm formation [23, 29, 30]. Although the regulatory mechanism for QS seems to be conserved in *Streptococci* [29], the knowledge about the crosstalk between *S. sanguinis* and *AAC*, leading to an enhanced biofilm formation, is still limited. Therefore, future works have to be done to shed light in the regulation of biofilm formation of *AAC* and *S. sanguinis* and typical microorganism involved in peri-implantitis [31].

Our results revealed that *AAC* increases the amount of *S. sanguinis* during biofilm formation on titanium surfaces. However, only *S. sanguinis* was quantified via qPCR, which means that no statement can be made about the amount of *AAC* within the biofilm. Thus, we cannot exclude whether the amount of *AAC* is changed under the tested conditions as well. Szafranski et al. already showed, that the cell number of *AAC* is not significantly increased in presence of *S. mutans* [23], but Sliepen et al.. demonstrated that the colonization of *AAC* on epethelium cells in presence of *S. sanguinis* can be inhibited [32]. We have studied biofilm formation on implants and not in connection with cell tissues. Therefore, we assume that only the biofilm of *S. sanguinis*, indicated by the increased amount of DNA, and not of *AAC* is affected during biofilm formation.

## Conclusions

We demonstrate that beside the known inducing effect of *AAC* on biofilm formation of *S. mutans* [23], this microorganism also leads to increased biofilm formation of *S. sanguinis* by a so far unknown mechanism. This shows that *AAC* has a broad spectrum for cell-cell communication within the oral microbiome and is therefore a decisive factor for biofilm formation on implant surfaces within the oral cavity.

Although *AAC* is discussed controversial as a typical representative of peri-implantitis, our experiments have shown that this microorganism can induce *Streptococci* in general, regardless of whether they are early or late colonizers, and that it can therefore be induced in the early stages of peri-implantitis. Therefore, the early biofilm formation in correlation with peri-implantitis seems to require the cross-talk of microorganism for a successful coloization.

Future experiments need to be performed to provide a deeper insight into the crosstalk of microorganisms within the oral microbiome and their influence on biofilm formation of surface structures.

## Data Availability

No datasets were generated or analysed during the current study.

## Abbreviations

qPCR: Quantitative polymerase chain reaction
R_a_: Average roughness
µm: Micrometer
mm: Millimeter
µl: Microliter
nm: Nanometer
°C: Celsius
h: Hour
rpm: Rounds per minute
ml: Milliliter
PCR: Polymerase chain reaction
DNA: Deoxyribonucleic acid
Ct: Cycle threshold
m: Meter
mN: Millinewton
